# Cytokinin Regulation of Source-Sink Relationships in Plant-Pathogen Interactions

**DOI:** 10.3389/fpls.2021.677585

**Published:** 2021-08-24

**Authors:** Kathryn E. McIntyre, Daniel R. Bush, Cristiana T. Argueso

**Affiliations:** ^1^Department of Agricultural Biology, Colorado State University, Fort Collins, CO, United States; ^2^Graduate Program in Cell and Molecular Biology, Colorado State University, Fort Collins, CO, United States; ^3^Department of Biology, Colorado State University, Fort Collins, CO, United States

**Keywords:** cytokinins, source-sink relationships, plant growth, plant yield, plant-pathogen interactions, carbon allocation, amino acid translocation

## Abstract

Cytokinins are plant hormones known for their role in mediating plant growth. First discovered for their ability to promote cell division, this class of hormones is now associated with many other cellular and physiological functions. One of these functions is the regulation of source-sink relationships, a tightly controlled process that is essential for proper plant growth and development. As discovered more recently, cytokinins are also important for the interaction of plants with pathogens, beneficial microbes and insects. Here, we review the importance of cytokinins in source-sink relationships in plants, with relation to both carbohydrates and amino acids, and highlight a possible function for this regulation in the context of plant biotic interactions.

## Introduction

Cytokinins are a group of plant hormones derived from adenine, classified by the presence of an isoprenoid or an aromatic chain at the N^6^ position of their adenine moieties (Mok and Mok, 2001). Although different compounds with cytokinin activity have been shown to regulate various physiological processes in plants, cytokinins are broadly described as growth promoting plant hormones. The first cytokinin discovered by Miller and Skoog in the 1950s, kinetin, was defined as a plant-derived chemical that could promote cell division (Miller et al., 1956). In a following study, it was demonstrated that kinetin, in combination with auxin, was responsible for promoting cell division and organ development from undifferentiated cells in culture (Skoog and Miller, 1957). While the study of cytokinins began in the middle of the 1900s, they are in fact an ancient hormone, being one of the first four hormones to emerge in photosynthetically capable organisms (Wang et al., 2015). Evolutionary studies indicate that the common ancestor of all land plants, charophytes, contains the genetic sequences of orthologs to known members the cytokinin signaling pathway (Wang et al., 2015). These data suggest that cytokinins had a role in plants as early as 450 million years ago. Today, cytokinins are known for their broad role in plant growth (Kieber and Schaller, 2018), and also roles in preventing senescence, as well regulation of biotic and abiotic stress tolerance (Argueso et al., 2009; Cortleven et al., 2019).

One important physiological response that has been classically associated with cytokinins is the regulation of source-sink relationships and nutrient allocation in plants. Shortly after cytokinins were discovered to have a role in cell division, a study in 1961 in *Nicotiana rustica* demonstrated that exogenous application of kinetin to leaves led to increased accumulation of the amino acid glycine to the area of hormone application (Mothes and Engelbrecht, 1961). Similarly, kinetin application to leaves of fava bean plants that had been unrooted was also shown to correlate with the movement of the amino acid alanine to the site of hormone application, which the researchers termed “mobilization” (Mothes and Engelbrecht, 1963). These early reports indicated that cytokinins could have a pivotal role in the allocation of amino acids in plants, with important consequences for plant growth.

In this review, we start by providing readers with an overview of the process of source-sink relationships in plants, and then proceed to highlight the evidence for a regulatory role for cytokinins in this important physiological process, starting with their first initial association and finishing with the most recent evidence. We conclude by pointing out some emerging evidence of the importance of this plant hormone as a regulator of nutrient availability in plant biotic interactions during disease susceptibility and promotion of plant immunity.

## Overview of Source-Sink Relationships in Plants

Photosynthesis leads to the production of reduced carbon products, also known as photoassimilates. Photoassimilates generated in the mesophyll cells, such as sucrose, oligosaccharides, and amino acids, are transported to other parts of the plant to maintain plant growth. Generally, the rate of photosynthetic activity and the accumulation of photoassimilates can be used to classify organs as sinks or sources. Sources are defined as photosynthetically active leaves that export photoassimilates to heterotrophic sink tissues that are dependent on imported sugars and amino acids for growth and development. Fully mature source leaves export as much as 80% of photoassimilate to sink tissues (Kalt-Torres et al., 1987). Sinks are defined as the opposite: an organ that is dependent on sugar and amino acid import to support growth and development. Sinks include young leaves, reproductive organs, and roots. Photosynthetic activity changes during the course of leaf development. Young leaves are sink organs that need to import photoassimilates from mature leaves to support growth and development (Geiger and Sheigh, 1993). As the immature leaf grows, it becomes photosynthetically active and eventually becomes an exporter of photoassimilates, through a process known as the sink-source transition (Turgeon, 1989). The relationship between source and sink organs has been the focus of intensive research because of its impact on plant growth and yield (White et al., 2016) and its potential for using transgenic approaches for modifying yield and/or nutritional quality (Yadav at al., 2015).

**Figure 1:**
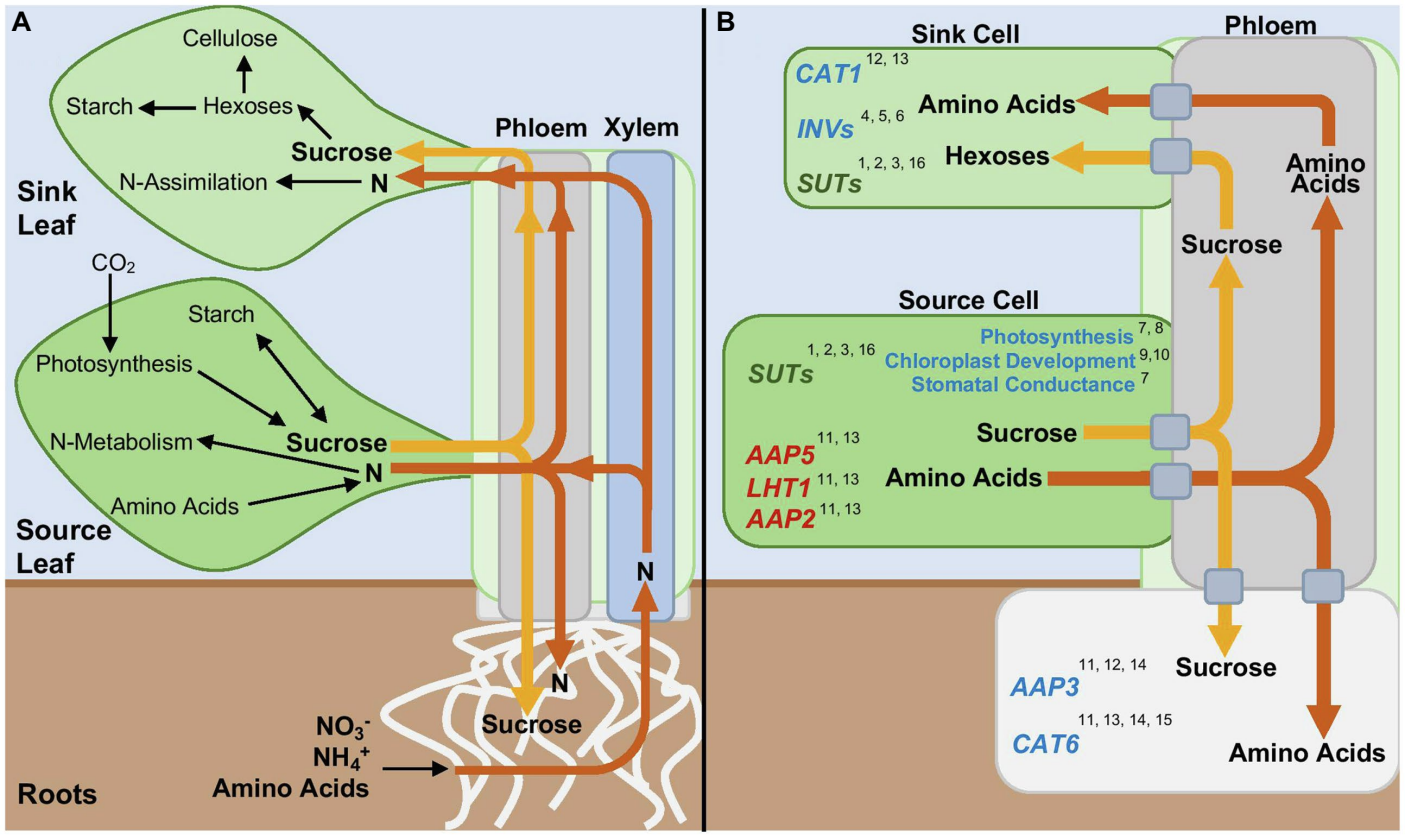
Source-Sink Relationships in Plants. **(A)** Whole plant movement of nitrogen containing compounds (N, orange arrows) and sugars (yellow arrows) between source and sink tissues. **(B)** Regulation by cytokinin of specific enzymes, transporters, and processes involved in source-sink relationships. Blue and red symbolize positive or negative regulation by cytokinin, respectively. Green symbolizes both a positive and negative regulation by cytokinin. Numbers correspond to references. References: 1. Harms et al., 1994; 2. Ninan et al., 2019; 3. Song et al., 2015; 4. Ehness and Roitsch, 1997; 5. Godt and Roitsch, 1997; 6. Yang et al., 2014; 7. Chernyad’ev, 2000; 8. Ahanger et al., 2018; 9. Boasson and Laetsch, 1969; 10. Okazaki et al., 2009; 11. Brenner et al., 2005; 12. Lee et al., 2007; 13. Kiba et al., 2011; 14. Kiba et al., 2005; 15. Yokoyama et al., 2007; and 16. Jian et al., 2016.

Sucrose is the end product of photosynthesis, and the primary sugar transported within plants. In source leaves, sucrose produced, from photosynthesis during the day or starch degradation occurring at night, is loaded into the phloem for transport to sinks (Figure 1A). Although this review focuses mostly on sucrose, as it is present in the phloem sap of all plant species, it should be noted that the phloem sap of some plant species also contains sugar alcohols and/or oligosaccharides from the raffinose family (Zimmermann and Ziegler, 1975; Noiraud et al., 2001). Depending on the anatomical connections of the plant species, loading of sucrose into the phloem can be achieved by three different loading mechanisms: symplastic, apoplastic, and polymer trapping (Braun et al., 2014). For the purposes of this review, we will focus on the apoplastic loading pathway, which is the predominant pathway used in most plant species, including the model plant species *Arabidopsis thaliana* (hereafter, Arabidopsis).

Apoplastic phloem loading in the leaf is mediated by a proton-sucrose symporter (Bush, 1993). Sucrose is transported out of mesophyll cells into the intercellular space by sucrose transporters known as SUGARS WILL EVENTUALLY BE EXPORTED TRANSPORTERS (SWEETs) (Chen et al., 2012). Once in the intercellular space, sucrose is then actively loaded into the phloem cells against a significant concentration gradient by proton-sucrose symporters, named SUCROSE TRANSPORTERS/CARRIERS (SUTs/SUCs) (Reinders et al., 2012; Zhang and Turgeon, 2018). Sucrose accumulates to molar levels in the leaf phloem thereby creating a high osmotic potential that draws in water. Since the phloem cells are surrounded by an inelastic cell wall, this creates high hydrostatic pressure that drives mass flow of solution to sink tissues where sucrose is released and used for growth, development or carbohydrate storage (Bush, 2020). There are two main mechanisms by which sucrose is moved into sink cells (Braun et al., 2014): (i) it is released into the intracellular space by SWEETs and then transported into the sink tissue by SUTs/SUCs (Weber et al., 1997) or (ii) it is released into intracellular space by SWEETs and then hydrolyzed into glucose and fructose by extracellular invertases (Ruan et al., 2010) followed by import into sink cells by proton/hexose symporters (HXTs) (Zhang et al., 2006; Hayes et al., 2007). Due to sucrose being the major form of carbon being translocated from source to sink (Fife et al., 1962; Turgeon, 1989), and starch being the main storage form of sucrose, the homeostasis of these two carbohydrates is essential for the regulation of their metabolism and allocation in plants (Smith and Stitt, 2007).

The production, storage, and movement of amino acids can also define organs as sinks or sources (Figure 1A) (Bush, 1999). Roots are the site of uptake of inorganic nitrogen (N) from the soil, in the form of nitrate or ammonium, a process regulated by transporters located in root epidermal hairs and root cortical and endodermal cells (Tegeder, 2014). However, some plant species are also able to take up organic N in the form of amino acids, depending on environmental and soil conditions (Nasholm et al., 2009; Tegeder and Rentsch, 2010; Bloom, 2015). The location of N assimilation, or the conversion of inorganic N into amino acids, varies among plant species (Masclaux-Daubresse et al., 2010). Nitrate taken up by the roots is primarily transported to the shoot prior to assimilation, while ammonium, due its toxic nature, is assimilated after uptake in the roots (Tegeder and Masclaux-Daubresse, 2018).

Transport of amino acids from the roots to the above ground areas of the plant occurs through the xylem, while translocation between source and sink organs occurs via the phloem (Tegeder and Masclaux-Daubresse, 2018) (Figure 1A). Once formed in source leaves or in roots, amino acids are loaded into the phloem and then unloaded into sinks tissues by amino acid transporters (Okumoto et al., 2004; Tegeder and Hammes, 2018). Also relevant to amino acid allocation is the process of plant senescence. During senescence leaf proteins are degraded, providing a large quantity of amino acids that is used for growth in other organs, a process known as amino acid remobilization. Amino acid remobilization also occurs through the action of amino acid transporters. In both Arabidopsis and *Brassica napus*, it has been shown through ^15^N tracing that senescing leaves are the primary source of N provided to sink tissues during the late vegetative phase or to flowers and seeds during the reproductive phase (Malagoli et al., 2005; Diaz et al., 2008; Lemaitre et al., 2008).

## Cytokinin Regulation of Source-Sink Relationships: Effect on Photosyntheses and Sucrose Transport

In many plant species, cytokinins positively affect photosynthetic rates (reviewed in Cherniad’ev, 2000). This effect is associated with increases in stomatal conductance and gas exchange, leading to higher photosynthetic rates and sucrose production (Ahanger et al., 2018, 2020). Cytokinins have also long been associated with an increase in chloroplast number per cell (Boasson and Laetsch, 1969), a process that has been coupled to the anti-senescence activity of this hormone. In Arabidopsis, this increase in chloroplast number per cell is facilitated by the transcriptional regulation of components of the chloroplast division machinery, which is mediated by the cytokinin-regulated transcription factor CYTOKININ RESPONSE FACTOR 2 (CRF2) (Okazaki et al., 2009). Because the number of chloroplasts within a cell can affect overall photosynthetic rates (Austin II and Webber, 2005; Xiong et al., 2017), cytokinin regulation of chloroplast number and their development may have a role in regulating source activity and strength and the availability of photoassimilates.

The photosynthetic activity of source tissues also changes, depending on the demand for photoassimilates by sinks (Paul and Foyer, 2001; Sonnewald and Fernie, 2018). When source leaves are shaded or removed by defoliation, the remaining source leaves display an increase in their rate of photosynthesis, compensating for the removed/shaded source leaves, responding to the rate of utilization of carbohydrates in sinks (Thorne and Koller, 1974; Peet and Kramer, 1980; McCormick et al., 2006). A study in tomato provided evidence that endogenous cytokinin levels could be responsible for altering the response of source leaves following defoliation. After defoliation, the increased photosynthesis levels observed in the remaining source leaves were positively correlated not only to increased levels of the cytokinin *trans*-zeatin riboside, but also to increased leaf expansion and decreased levels of sugar export. This study suggests that the increased cytokinin concentration in the source leaves caused higher photosynthetic activity, resulting in sugar production that was used for leaf expansion, instead of transport to sinks, which ultimately increased the strength of the source tissue (Glanz-Idan et al., 2020).

As previously mentioned, one method of sugar uptake in sinks following unloading from the phloem is through the activity of extracellular invertases, located within the cell wall. Extracellular invertases catalyze the hydrolysis of sucrose into glucose and fructose (Ruan, 2014), a process that can influence the sink strength (Ho, 1988; Herbers and Sonnewald, 1998; Sturm and Tang, 1999; Lemoine et al., 2013). This process seems to be regulated by cytokinins: Exogenous application of the cytokinin *trans-*zeatin can increase the expression of extracellular invertases (Godt and Roitsch, 1997; Lara et al., 2004) and in a detached leaf assay, cytokinins can prevent senescence and maintain sink strength through regulation of the activity of extracellular invertases (Lara et al., 2004).

Cytokinins may also have a role in sucrose transport from source to sink organs by regulating the expression of *SWEET* and *SUT/SUC* transporters (Table 1). In potato, expression of the sucrose transporter *StSUT1* was shown to be induced in mature leaves following exogenous treatment with the cytokinin benzyladenine (BA) (Harms et al., 1994) and in *Brassica napus* expression of *BnSUT*s and *BnSWEET*s was increased after exogenous application of BA to leaves (Jian et al., 2016). Endogenous levels of cytokinins also regulate *SUC/SUT/SWEET* expression. In peas, the content of several cytokinin species in source leaves is correlated with the increase in expression of genes encoding SWEETs and SUTs (Ninan et al., 2019). Developing fruits and seeds are considered major sink tissues. As seeds develop, the walls of siliques in *B. napus* show an increase in expression of *BnSUTs*, which is also correlated with an increased expression in genes responsible for cytokinin biosynthesis (Song et al., 2015). Although the correlation in the expression of sugar transporters and cytokinin content is not necessarily causative, evidence exists for a functional role of this regulation in sugar transport: Application of the cytokinins BA, kinetin, or *trans-*zeatin to *Chenopodium rubrum* suspension cells did not only lead to increased expression of the hexose transporter genes *CST2* and *CST3*, but also to increased uptake of ^14^C-labeled glucose from the cell suspension media as compared to untreated cells (Ehness and Roitsch, 1997).

**Table 1:** Transcriptional regulation by cytokinins of genes encoding invertases, sugar, and amino acid transporters in various plant species.

Species	Gene	Gene description	CK regulation
Carbohydrate-related			
*Zea mays*	*IVR1*	Vacuolar invertase	+
	*IVR2*	Vacuolar invertase	+
*Chenopodium rubrum* *Solanum lycopersicum*	*CIN1*	Extracellular invertase	+
	*LIN6*	Extracellular invertase	+
Sucrose transport			
*Arabidopsis thaliana*	*CST2*	Hexose transporter	+
	*CST3*	Hexose transporter	+
*Pisum sativum*	*PsSW12*	Sucrose transporter	−
	*PsSUT1*	Sucrose transporter	−
	*PsSUT2*	Sucrose transporter	−
*Solanum tuberosum*	*StSUT1*	Sucrose transporter	+
*Brassica napus*	*BnSUT1*	Sucrose transporter	+
	*BnSUT2*	Sucrose transporter	+
	*BnSUT3*	Sucrose transporter	+
	*BnSUT4*	Sucrose transporter	+
Amino acid transporter			
*Arabidopsis thaliana*	*AAP2*	Amino acid transporter	−
	*AAP3*	Amino acid transporter	+
	*AAP5*	Amino acid transporter	−
	*CAT1*	Amino acid transporter	+
	*CAT6*	Amino acid transporter	+
	*LHT1*	Amino acid transporter	−
*Brassica napus*	*BnAAP1*	Amino acid transporter	−
	*BnAAP2*	Amino acid transporter	−
	*BnAAP4*	Amino acid transporter	−
	*BnAAP5*	Amino acid transporter	−
	*BnAAP6*	Amino acid transporter	−
	*BnAAP7*	Amino acid transporter	−
	*BnAAP8*	Amino acid transporter	−
*Pisum sativum*	*PsAAP3*	Amino acid transporter	−
	*PsAAP6a*	Amino acid transporter	−
	*PsAAP7b*	Amino acid transporter	−
*Oryza sativa*	*OsAAP1*	Amino acid transporter	+
	*OsLHT1*	Amino acid transporter	+

## Cytokinin Regulation of Source-Sink Relationships: Effect on Amino Acid Transport

The first evidence of a potential role for cytokinins in source-sink relationships came from studies on the movement of amino acids in response to kinetin application to plants. Mothes and Engelbrecht showed that when kinetin was applied to detached leaves of *Nicotiana rustica*, ^14^C-labeled glycine migrated to the site of kinetin application (Mothes and Engelbrecht, 1961). A similar experiment in unrooted seedlings of fava beans also showed translocation of ^14^C-labeled alanine to sites of kinetin application. However, if plants were rooted, ^14^C-labeled alanine migration to sites of cytokinin application was diminished, with more ^14^C-labeled alanine being mobilized to roots (Mothes and Engelbrecht, 1963). Given that roots are sites of cytokinin biosynthesis (Miyawaki et al., 2004), these experiments showed a direct link between amino acid mobilization and cytokinin content. A similar effect of cytokinin on amino acid mobilization was shown in other plant species, including monocot species, such as oats (Gunning and Barkley, 1963), as well as beans and maize plants (Leopold and Kawase, 1964). Importantly, non-proteinogenic amino acids, such as α-aminoisobutyric acid, are also mobilized to sites of cytokinin application, indicating that the effect of cytokinin is not due to an increased need of amino acids for protein synthesis, but on amino acid translocation *per se* (Mothes and Engelbrecht, 1961).

The relationship between cytokinin and amino transporters has been examined mostly at the level of regulation of gene expression of amino acid transporter genes, such as those from the family amino acid permeases (AAP), lysine and histidine transporters (LHT), and cationic amino acid transporters (CAT). Application of cytokinin increases the expression of *AAP3* (Brenner et al., 2005; Kiba et al., 2005)*, CAT1* (Kiba et al., 2005), and *CAT6* (Brenner et al., 2005; Kiba et al., 2005; Yokoyama et al., 2007), and decreases expression of *AAP2, AAP5,* and *LHT1* (Brenner et al., 2005; Kiba et al., 2011; Figure 1B and Table 1). Further, transgenic plants with reduced cytokinin signaling display decreased expression of *CAT1* and *AAP3* (Lee et al., 2007).

The different effects that cytokinins have on the levels of expression of genes encoding amino acids transporters are likely explained by the differences in the ability of these transporters in facilitating the movement of specific amino acids, as well as their distinct expression patterns in different tissues within plants. In general, those that are upregulated by cytokinin tend to be expressed in sink organs, such as roots and flowers (Okumoto et al., 2004; Su et al., 2004; Tegeder et al., 2011), and some of them, such as *CAT6,* which is expressed in root tips, have been shown genetically to function in supplying sink cells with amino acids (Hammes et al., 2006). *AAP2, AAP5*, and *LHT1*, on the other hand, are downregulated by cytokinins, and their function and expression patterns seem to be associated with phloem loading in sources. *aap2* mutants display reduced amino acid content in the phloem, thus suggesting a function in phloem loading (Zhang et al., 2010). *AAP5* expression is observed in source leaves, but not sink leaves (Fischer et al., 1995, 2002). Similarly, *LHT1* expression is observed mostly in source organs and is likely involved in the transport of amino acids between mesophyll cells and the xylem (Ehness and Roitsch, 1997; Hirner et al., 2006). Experiments outside of the model plant species Arabidopsis have also provided evidence of the association between cytokinins and regulation of the expression of genes involved in amino acid transport (Song et al., 2015; Ninan et al., 2019; Zhu et al., 2020). In addition to these observed changes in expression of amino acid transporter genes in response to cytokinins, corresponding changes in amino acid translocation are also observed. A study in wheat showed that application of the cytokinin BA to source leaves dramatically decreases the content of amino acids present in the phloem, thus suggesting a function in decreasing phloem loading (Criado et al., 2009).

Finally, amino acid and sugar metabolism are connected in several ways, including through the non-proteinogenic amino acid γ-amino butyric acid (GABA). GABA is synthesized through the GABA shunt pathway, named as such because it bypasses two steps of the tricarboxylic acid (TCA) cycle that is essential to the catabolism of sugars for cellular respiration (Bouche and Fromm, 2004). GABA production through the GABA shunt results from the decarboxylation of the amino acid glutamate, and GABA catabolism leads to the production of succinate that then enters the TCA cycle. Thus, GABA connects amino acid production and sugar utilization. Cytokinins have not been directly associated with GABA production, but plants with increased levels of the cytokinin *trans*-zeatin accumulate GABA at higher levels and that are correlated to increased drought tolerance (Merewitz et al., 2012).

## Cytokinins and Source-Sink Relationships in the Outcome of Plant Biotic Interactions

Although cytokinins are broadly known as plant hormones involved in the regulation of plant growth, in the last few decades, their involvement in plant-pathogen interactions has become evident (reviewed in Albrecht and Argueso 2017; Akhtar et al., 2020). Similarly, a growing body of evidence has accumulated that indicates an important role for nutrient partitioning in creating metabolic conditions that favor or restrict pathogen growth in plant hosts. In the paragraphs that follow, we highlight a role for source-sink relationships in plant biotic interactions, with emphasis on plant-pathogen interactions, and suggest a function for the plant hormone cytokinin in the regulation of this process.

## May i Offer you Something to Eat? Cytokinins and Source-Sink Relationships in Disease Susceptibility

After successful invasion of the host, plant pathogens use effectors (secreted proteins, secondary metabolites, or nucleic acids of pathogen origin) to colonize the host and create host metabolic conditions that are favorable for pathogens, leading to plant susceptibility. Such metabolic conditions include the manipulation of plant metabolism to feed the growing number of pathogens that starts to multiply on the infected plant tissue. While some examples exist of studies on the importance of source-sink relationships in the association of plants with necrotrophic pathogens (Lemonnier et al., 2014; Veillet et al., 2016), which are those that kill plant host cells for their nutrition, the majority of studies has focused on the association of plants with biotrophic pathogens, given the dependency of such pathogens on living plant cells as their source of nutrients.

The role of cytokinins in increasing plant susceptibility to pathogen attack has been well documented. This effect is most commonly seen when lower concentrations of cytokinins are applied to plants prior to pathogen infection (Babosha, 2009; Argueso et al., 2012; Hann et al., 2014). However, in addition to plants, several other organisms can also produce cytokinins or manipulate cytokinin metabolism and/or signaling in plants, including parasitic plants, insects, and plant-associated microbes (reviewed in Spallek et al., 2018). Such microbes include plant pathogenic microbes, able to cause disease on plants, and also beneficial ones, whose association with plants results in enhanced plant growth and protection from disease. By manipulating cytokinin metabolism and/or signaling in plants, such organisms can also potentially regulate plant susceptibility, through manipulation of host physiology.

For the most part, the majority of interactions involving cytokinin production or manipulation by pathogens involves the creation of sink tissues for pathogen nutrition, accompanied by plant developmental changes, such as galls, tumors, and knots, which are usually noted as disease symptoms. Such developmental changes are associated with one of the primary functions of cytokinins, namely, cell division. However, as a secondary effect, these regions of cell proliferation and growth in fact create new sink tissues and thus alter the balance of source-sink relationships within the plant. A classic example of a plant pathogen that utilizes biosynthesis of cytokinins to create new sink tissues is *Agrobacterium tumefaciens*, the causal agent of crown gall disease. Agrobacterium cells carry a *Tumor-inducing* (*Ti*) plasmid containing the cytokinin biosynthesis gene *trans-zeatin synthesizing* (*tzs*), which is inserted into the plant genome to lead to cytokinin biosynthesis in plant cells (Liu and Kado, 1979; Akiyoshi et al., 1984, 1987; Kutáček and Rovenská, 1991; Lee et al., 2009; Hwang et al., 2010). Along with bacterial-induced auxin biosynthesis, the induction of cytokinin biosynthesis by Agrobacterium results in cell proliferation and the formation of galls. Metabolites needed for gall tumor growth are then rerouted from host plant source leaves to the crown gall tumor, which becomes a strong sink (reviewed in Gohlke and Deeken, 2014).

Another root gall-forming plant pathogen, the obligate biotroph *Plasmodiophora brassicae,* causes clubroot disease in cruciferous plants. The genome of *P. brassicae* contains two cytokinin biosynthesis genes (Schwelm et al., 2015) that were shown to contribute, albeit in a small manner, to the overall cytokinin content in infected tissue (Malinowski et al., 2016). Infection of Arabidopsis by *P. brassicae* alters carbohydrate metabolism of the host, resulting in increased sugar and starch content at the site of infection (Williams et al., 1968; Evans and Scholes, 1995; Brodmann et al., 2002). This carbohydrate mobilization was suggested to be due to high localized concentrations of cytokinins, which create a carbohydrate sink (Dekhuijzen, 1980) mediated by the sugar transporters SWEET11 and SWEET12 (Walerowski et al., 2018). However, decreased disease symptoms were seen after *P. brassicae* infection of the cytokinin biosynthesis mutant ipt1;3;5;7 indicating that the pathogen-derived cytokinins are not sufficient to create a sink (Malinowski et al., 2016). The gall-forming bacteria, *Rhodococcus fascians,* is also known to produce cytokinins as part of its virulence strategy (Stes et al., 2013). Pea plants infected with *R. fascians* show an increase in chlorophyll content, bacterial produced cytokinins, and endogenous plant-derived cytokinins in infected cotyledons (Depuydt et al., 2008; Dhandapani et al., 2017). Moreover, this is accompanied by an increase in expression of *PsCWINV*, *PsSUT*, and *PsSW* (*SWEET*) sugar transporter genes (Dhandapani et al., 2017), suggesting that during infection cytokinins may play a role in creating and maintaining infection sites as sinks tissues. A similar relationship is seen between Arabidopsis and the cyst nematode *Heterodera schachtii*. Upon invading plant roots, this species of nematode induces the formation of specialized structures named syncytia. *H. schachtii* was shown to produce and secrete cytokinins during infection of plant cells, and silencing of the *HsIPT* gene encoding the nematode cytokinin biosynthetic enzyme led to decreased syncytia size and decreased nematode size (Siddique et al., 2015). Given that syncytia are essential sites for juvenile feeding, these results implicate cytokinin as a nematode factor that is necessary to establish nematode feeding sites as sinks, promoting pathogen growth. Further, Arabidopsis amino acid transporters *AAP3* and *AAP6*, which belong to a class of amino acid transporters known to be transcriptionally regulated by cytokinins (Brenner et al., 2005; Kiba et al., 2005; Lee et al., 2007), are necessary for infection of Arabidopsis plants by the root-knot nematode *Meloidogyne incognita,* indicating that successful colonization is dependent on amino acid transport to the sites of infection (Marella et al., 2013), in a process that may be mediated by cytokinins.

**Figure 2:**
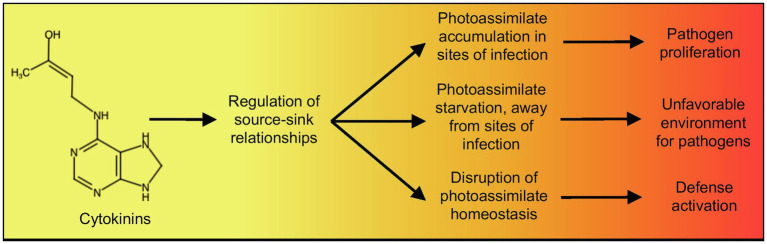
Summary of the proposed effect of cytokinins, through their role in the regulation of source-sink relationships, on the outcome plant-pathogen interactions. Chemical structure created using http://chem-space.com.

Plant-pathogen associations involving cytokinins can also contribute to changes in source-sink relationships without the activation of cell division to create sinks. Such an effect of cytokinins can be seen in the formation of green islands, small areas of live and green leaf tissue surrounded by yellow, senescing tissue, in plants infected with biotrophic fungi (Bushnell, 1967). Green islands have an increased cytokinin content within the green areas (López-Carbonell et al., 1998), which also display increased rates of photosynthesis in comparison with the surrounding senescing tissue (Walters et al., 2008), as well as increased levels of amino acids, sugars, and starch (Raggi, 1974, 1976; Angra and Mandahar, 1991; Angra-Sharma and Mandahar, 1993). These physiological changes in green islands are reminiscent of cytokinin-mediated changes in source-sink relationships mediated by cell wall invertases (Lara et al., 2004), and likely function to maintain these sites as sinks suitable for biotrophic pathogen growth. *Magnaporthe oryzae,* the rice blast fungus, also has the ability to produce cytokinin (Chanclud et al., 2016). *M. oryzae* mutants in the cytokinin biosynthetic gene *CKS1* have reduced virulence and are impaired in their ability to multiply *in planta,* but not *in vitro*, implicating pathogen nutrition through host-derived mechanisms in the reduced virulence phenotype of the mutant (Chanclud et al., 2016). This cytokinin-dependent virulence was associated with the allocation of sugars and amino acids (namely, aspartate and glutamate) to the sites of infection (Chanclud et al., 2016), thus suggesting a function for cytokinin in acting to change source-sink relationships and nutrient allocation in sites of infection, promoting conditions for pathogen multiplication (Figure 2).

On a final note, it is important to mention that beneficial microbes also utilize cytokinins in their association with plants to manipulate source-sink relationships and plant growth. In one of the most well-studied examples, cytokinins are essential for nodule formation during the interaction between Rhizobia bacteria and legume plants. In such interactions, plants redirect photoassimilates, mainly in the form of sucrose, to the bacteria, in exchange for organic nitrogen (Kennedy, 1966; Bergersen and Turner, 1967; Kouchi and Yoneyama, 1984). Root nodules can then be classified as sink organs, which require cytokinin for their formation. In *Medicago truncatula*, this requirement for cytokinins is mediated by the ABC transporter ABCG56, which functions as a cytokinin exporter and is required for nodule formation (Jarzyniak et al., 2021). In addition, plant-derived cytokinins are also needed for the activity of certain volatile organic compounds produced by beneficial rhizobacteria, which induce plant growth. This cytokinin-dependent, rhizobacteria-mediated plant growth is associated with increased photosynthesis and nutrient acquisition, thus linking it to source-sink relationships (Ryu et al., 2003; Zhang et al., 2008; Gutiérez-Luna et al., 2010; Vacheron et al., 2013; Ditengou et al., 2015; Cordovez et al., 2018).

## Starving the Attacker and Sounding the Alarm: Cytokinins and Source-Sink Relationships in Defense Responses

In addition to a role in creating sink tissues for pathogen nutrition, accumulating evidence also exists for a role of source-sink relationships in defense responses as well. In Arabidopsis, regulation of the sugar transporter STP13 leads to altered susceptibility to pathogens (Yamada et al., 2016). *STP13* is expressed in leaf tissues after infection with the bacterial pathogen *Pseudomonas syringae* pv. *tomato*. Its transport activity was shown to be suppressed via phosphorylation by a protein complex composed of the extracellular immune receptor FLS2 (FLAGELLIN SENSITIVE 2) and its associated kinase BIK1 (BRASSINOSTEROID INSENSITIVE 1). Thus, upon perception of pathogen presence by the FLS2/BIK1 complex, plants diminish STP13 activity, effectively halting sugar transport to the apoplast and preventing pathogen feeding and multiplication (Yamada et al., 2016). STP13 is also important for resistance to the necrotrophic pathogen *Botrytis cinerea*, although it is unknown whether the regulatory mechanisms cited above also apply (Lemonnier et al., 2014).

Other examples of source-sink relationships being modified for defense responses to pathogens, rather than pathogen feeding, include the genes encoding proteinaceous invertase inhibitors. Proteinaceous invertase inhibitors are endogenous plant signals for invertase regulation in plants. In response to *Pseudomonas syringae* pv. *tomato* DC3000 infection, the expression of genes encoding these invertase inhibitors in Arabidopsis is downregulated, a fact that has always been interpreted as manipulation of plant metabolism by the pathogen to increase glucose and fructose availability for pathogen nutrition (Bonfig et al., 2006). However, the activity of these invertase inhibitors has been shown to in fact increase in infected resistant plants, thus functioning as a defense mechanism to prevent the pathogen from cleaving sucrose for its nutritional needs (Bonfig et al., 2010). Finally, the sugar transporters SUT1 and SUT2 in tomato have also been connected to defense responses in plants. *SUT1* and *SUT2* expression are downregulated during the infection of tomato plants with *Candidatus Phytoplasma solani*, an obligate biotrophic bacterial pathogen that inhabits host phloem cells (De Marco et al., 2021). Antisense analyses of *SUT1* and *SUT2* genes in tomato showed that absence of SUT1 and SUT2 function decreases susceptibility of tomato plants to this pathogen, without compromising plant growth, and at the same time increasing the expression of defense genes (De Marco et al., 2021), thus connecting source-sink relationships to defense activation. While a function for cytokinins in the control of source-sink relationships for pathogen nutritional deprivation has not yet been demonstrated, the general importance of this plant hormone in the physiological processes cited above makes it a likely candidate for such regulatory action.

In further agreement with a general role for source-sink relationships in defense is the fact that not only changes in sugar allocation, but also changes in amino acid allocation, lead to altered susceptibility to pathogens. Mutations or overexpression of genes encoding amino acid transporters can also lead to decreased susceptibility to pathogens. This is the case, for example, of the cytokinin-regulated amino acid transporter gene *LHT1*. *lth1* mutants display decreased susceptibility to *Pseudomonas syringae* p.v. *tomato*, as well the hemibiotrophic fungus *Colletotrichum higginsianum* and the biotrophic fungus *Golovinomyces cichoracearum* (Liu et al., 2010). Overexpression of the gene encoding the amino acid transporter *CAT1,* whose expression is also transcriptionally regulated by cytokinins (Kiba et al., 2005), leads to a decrease in susceptibility to *Pseudomonas syringae* p.v. *tomato* (Yang et al., 2014). Most recently, a mutation in the gene encoding the amino acid transporter USUALLY MULTIPLE ACIDS MOVE IN AND OUT 36 (UMAMIT 36) was shown to confer resistance to the oomycete *Phytophthora parasitica* (Pan et al., 2016), and overexpression of *UMAMIT 14* was shown to decrease susceptibility of Arabidopsis to another oomycete, *Hyaloperonospora arabidopsidis* (Besnard et al., 2021). What is interesting about the examples cited above is that the decreased susceptibility phenotypes of the lines with altered amino acid transporter genes are also accompanied by an increase in the endogenous levels of the defense hormone salicylic acid (SA), and elevated basal levels of the known SA defense marker gene *PATHOGENESIS-RELATED-1* (*PR-1*). Thus, the decrease in pathogen susceptibility is likely not due to altered amino acid transport leading to nutritional deprivation but is in fact due to activation of plant defense pathways and responses.

Amino acids are directly linked to the production of secondary metabolites with important roles in defense, such as glucosinolates and camalexins (derived from tryptophan), SA biosynthesis (derived from phenylalanine), and the biosynthesis of a primer of defense responses, pipecolic acid (derived from lysine). Therefore, it would be reasonable to conclude that these genetic alterations on amino acid transporter genes lead to changes in the cellular amino acid pool, with consequences to the biosynthesis of defense compounds and defense activation. Counterarguments to this amino acid pool hypothesis are several: (i) the fact that the amino acid transporter genes linked to altered pathogen responses are not directly linked to transport of the particular amino acids necessary for the corresponding defense compounds; (ii) that the resistance observed seems to be broad spectrum, and not associated with the effect of a particular defense compound; (iii) that increased levels of GABA, the non-proteinogenic amino acid involved in connecting N and C metabolism, are also associated with abiotic stress tolerance and resistance to pests (Seifikalhor et al., 2019; Tarkowiski et al., 2020); and (iv) and most importantly, that alterations in sugar transport and signaling also seem to activate defense responses in a similar manner to changes in amino acid homeostasis (Gebauer et al., 2017; De Marco et al., 2021). Such counterarguments favor another hypothesis, where cellular metabolic alterations may lead to the activation of defense responses, through a mechanism similar to metabolic priming. Priming is an activated state where plants are able to deploy stronger and faster defenses, resulting in enhanced pathogen protection (reviewed in Mauch-Mani et al., 2017), and the idea of metabolic priming for defense responses has recently been further investigated (Liu et al., 2010; Schwachtje et al., 2018, 2019).

The concept of metabolic priming shares remarkable similarities with the effect of cytokinins on plants. When applied in high concentrations to plants cytokinins can also lead to reduced susceptibility to a broad spectrum of pathogens (reviewed in Akhtar et al., 2020). This is accompanied by the increased production of antimicrobial compounds, such as phytoalexins (Ko et al., 2010; Grosskinsky et al., 2011), and also the production of reactive oxygen species (ROS) and increased defense gene expression, in a manner that is dependent on the defense hormones SA (Choi et al., 2010; Argueso et al., 2012; Naseem et al., 2012) and jasmonic acid (Gupta et al., 2020). Of note, similarly to what happens in defense priming, these responses to cytokinin only happen after pathogen detection. Therefore, cytokinins do not directly activate responses; rather, they trigger physiological conditions that potentiate defense.

The two hypotheses mentioned above, namely, changes in photoassimilate availability altering production of defense compounds or changes in photoassimilate availability altering cellular metabolic stress leading to priming, are not mutually exclusive. Both hypotheses could be parts of an integrated plant defense response involving the regulation of source-sink relationships, coordinated by the plant growth hormone cytokinin (Figure 2). In this context, cytokinin levels, through their general effect on source-sink relationships, would serve as a signal for changes in cellular and organismal metabolism that would activate defense. Such a mechanism would likely be beneficial to plants, as it would provide a way to connect defense activation to photoassimilate production, depending on fluctuating environmental conditions.

## Conclusions and Perspectives

Because plants are sessile organisms, their ability to effectively respond to environmental change is vital to their survival. To maintain proper growth and development, plants have adapted response mechanisms to regulate photosynthetic ability and photoassimilate partitioning, depending on environmental conditions, such as light intensity, temperature, and water availability. Just like other plant hormones that act on the regulation of cell expansion or cell division, cytokinins have long been associated with promotion of plant growth. In the case of cytokinins, the ability to promote greening and increasing photosynthesis rates is likely also involved in its stimulation of plant growth and yield, as this is centrally linked to the generation of more photoassimilates for plant growth. Further, how these photoassimilates are distributed in the plant are just as important for plant growth and yield, and it is in this aspect that the regulation by cytokinins of source-sink relationships plays a significant role, so much so that genes involved in aspects of cytokinin metabolism and signaling have been a frequent target of crop breeding programs centered on yield improvement (White et al., 2016). Because breeding programs target increased yields in different parts of the plant (seed, fruits, and vegetative organs) depending on the crop, the role of cytokinins in regulating sugar and amino acid transporters with tissue-specific patterns of expression may be of particular interest and importance.

Similarly, response to pathogen attack also requires complex responses by plants. To do so, plants have evolved sophisticated perception and signaling strategies, often mediated by plant hormones, including cytokinins. Timing and degree of defense activation must be tightly controlled, as insufficient defense responses could lead to host death, whereas excessive defense may result in inhibition of plant growth (reviewed in Albrecht and Argueso, 2017). Maintenance of balanced source-sink relationships is therefore vital to sustain growth while ensuring proper defense response against the pathogen. Evidence for the importance of this balanced response comes from the fact that pathogens have developed mechanisms of manipulation of source-sink relationships, in order to obtain nutrients for growth and multiplication. As it is common in the always evolving arms race between plants and pathogens, plants have also evolved ways to manipulate these source-sink relationships for defense purposes, and there is evidence that both processes may be partly regulated by cytokinins. Given the negative effect of plant pathogens on plant growth and yield, and the importance of photoassimilate partitioning to plant susceptibility and resistance, investigating the role of cytokinin-mediated source-sink relationships in the context of plant-pathogen interactions may provide new avenues not only for yield improvement, but also for pathogen resistance.

## Author Contributions

KM wrote the manuscript and prepared the figures. CA wrote and edited the manuscript and edited the figures. DB edited the manuscript. All authors contributed to the article and approved the submitted version.

## Conflict of Interest

The authors declare that the research was conducted in the absence of any commercial or financial relationships that could be construed as a potential conflict of interest.

## Publisher’s Note

All claims expressed in this article are solely those of the authors and do not necessarily represent those of their affiliated organizations, or those of the publisher, the editors and the reviewers. Any product that may be evaluated in this article, or claim that may be made by its manufacturer, is not guaranteed or endorsed by the publisher.

## References

[ref1] Ahanger M. A. Alyemeni M. N. Wijaya L. Alamri S. A. Alam P. Ashraf M.. (2018). Potential of exogenously sourced kinetin in protecting *Solanum lycopersicum* from NaCl-induced oxidative stress through up-regulation of the antioxidant system, ascorbate-glutathione cycle and glyoxalase system. PLoS One 13:9. 10.1371/journal.pone.0202175 PMC6122799 30180173

[ref2] Ahanger M. A. Aziz U. Sahli A. A. Alyemeni M. N. Ahmad P. (2020). Combined kinetin and spermidine treatments ameliorate growth and photosynthetic inhibition in *Vigna angularis* by up-regulating antioxidant and nitrogen metabolism under cadmium stress. Biomol. Ther. 10:147. 10.3390/biom10010147, PMID: 31963299 PMC7022836

[ref3] Akhtar S. S. Mekureyaw M. F. Pandey C. Roitsch T. (2020). Role of cytokinins for interactions of plants with microbial pathogens and pest insects. Front. Plant Sci. 10:6. 10.3389/fpls.2019.01777 PMC7042306 32140160

[ref4] Akiyoshi D. E. Klee H. Amasino R. M. Nester E. W. Gordon M. P. (1984). T-DNA of *Agrobacterium-tumefaciens* encodes an enzyme of cytokinin biosynthesis. Proc. Natl. Acad. Sci. U. S. A. 81, 5994–5998. 10.1073/pnas.81.19.5994 6091129 PMC391845

[ref5] Akiyoshi D. E. Regier D. A. Gordon M. P. (1987). Cytokinin production by *Agrobacterium* and *Pseudomonas* spp. J. Bacteriol. 169, 4242–4248. 10.1128/jb.169.9.4242-4248.1987, PMID: 3624204 PMC213736

[ref6] Albrecht T. Argueso C. T. (2017). Should I fight or should I grow now? The role of cytokinins in plant growth and immunity and in the growth-defence trade-off. Ann. Bot. 119, 725–735. 10.1093/aob/mcw211, PMID: 27864225 PMC5379597

[ref7] Angra R. Mandahar C. L. (1991). Pathogenesis of barley leaves by *Helminthosporium teres* I: green island formation and the possible involvement of cytokinins. Mycopathologia 114, 21–27. 10.1007/BF00436687

[ref8] Angra-Sharma R. Mandahar C. L. (1993). Involvement of carbohydrates and cytokinins in pathogenicity of *Helminthosporium carbonum*. Mycopathologia 121, 91–99. 10.1007/BF01103576

[ref9] Argueso C. T. Ferreira F. J. Epple P. To J. P. Hutchison C. E. Schaller G. E.. (2012). Two-component elements mediate interactions between cytokinin and salicylic acid in plant immunity. PLoS Genet. 8:1. 10.1371/journal.pgen.1002448 PMC3266875 22291601

[ref10] Argueso C. T. Ferreira F. J. Kieber J. J. (2009). Environmental perception avenues: the interaction of cytokinin and environmental response pathways. Plant Cell Environ. 32, 1147–1160. 10.1111/j.1365-3040.2009.01940.x, PMID: 19183294

[ref11] Austin J. II Webber A. N. (2005). Photosynthesis in *Arabidopsis thaliana* mutants with reduced chloroplast number. Photosynth. Res. 85, 373–384. 10.1007/s11120-005-7708-x 16170638

[ref12] Babosha A. V. (2009). Regulation of resistance and susceptibility in wheat-powdery mildew pathosystem with exogenous cytokinins. J. Plant Physiol. 166, 1892–1903. 10.1016/j.jplph.2009.05.014, PMID: 19592133

[ref13] Bergersen F. J. Turner G. L. (1967). Nitrogen fixation by the bacteriod fraction of breis of soybean root nodules. Biochim. Biophys. Acta Biochim Biophys Acta. 141, 507–515. 10.1016/0304-4165(67)90179-1 6069173

[ref14] Besnard J. Sonawala U. Maharjan B. Collakova E. Finlayson S. A. Pilot G.. (2021). Increased expression of UMAMIT amino acid transporters results in activation of salicylic acid dependent stress response. Front. Plant Sci. 11:606386. 10.3389/fpls.2020.606386 33574824 PMC7870477

[ref15] Bloom A. J. (2015). The increasing importance of distinguishing among plant nitrogen sources. Curr. Opin. Plant Biol. 25, 10–16. 10.1016/j.pbi.2015.03.002, PMID: 25899331

[ref16] Boasson R. Laetsch W. M. (1969). Chloroplast replication and growth in tobacco. Science 166, 749–751. 10.1126/science.166.3906.749 17776764

[ref17] Bonfig K. B. Gabler A. Simon U. K. Luschin-Ebengreuth N. Hatz M. Berger S.. (2010). Post-translational derepression of invertase activity in source leaves via down-regulation of invertase inhibitor expression is part of the plant defense response. Mol. Plant 3, 1037–1048. 10.1093/mp/ssq053, PMID: 20833735

[ref18] Bonfig K. B. Schreiber U. Gabler A. Roitsch T. Berger S. (2006). Infection with virulent and avirulent *P. syringae* strains differentially affects photosynthesis and sink metabolism in *Arabidopsis* leaves. Planta 225, 1–12. 10.1007/s00425-006-0303-3, PMID: 16807755

[ref19] Bouche N. Fromm H. (2004). GABA in plants: just a metabolite? Trends Plant Sci. 9, 100–115. 10.1016/j.tplants.2004.01.006 15003233

[ref20] Braun D. M. Wang L. Ruan Y. L. (2014). Understanding and manipulating sucrose phloem loading, unloading, metabolism, and signalling to enhance crop yield and food security. J. Exp. Biol. 65, 1713–1735. 10.1093/jxb/ert416 24347463

[ref21] Brenner W. G. Romanov G. A. Kollmer I. Burkle L. Schmulling T. (2005). Immediate-early and delayed cytokinin response genes of *Arabidopsis thaliana* identified by genome-wide expression profiling reveal novel cytokinin-sensitive processes and suggest cytokinin action through transcriptional cascades. Plant J. 44, 314–333. 10.1111/j.1365-313X.2005.02530.x, PMID: 16212609

[ref22] Brodmann A. Schuller A. Ludwig-Miller J. Aeschbacher R. A. Wiemken A. Boller T.. (2002). Induction of trehalase in *Arabidopsis* plants infected with the trehalose-producing pathogen *Plasmodiophora brassicae*. Mol. Plant-Microbe Interact. 15, 693–700. 10.1094/MPMI.2002.15.7.693, PMID: 12118885

[ref142] Bush D. R. (1993). Proton-coupled sugar and amino acid transporters in plants. Ann. Rev. Plant. Physiol. Plant. Mol. Biol. 44, 513–542. 10.1146/annurev.pp.44.060193.002501

[ref143] Bush D. R. (1999). “Amino acid transport,” in Plant Amino Acids: Biochemistry and Biotechnology. ed. Singh B. K. (NY: Marcel Dekker), 305–318.

[ref144] Bush D. R. (2020). Identifying the pathways that control resource allocation in higher plants. Proc. Natl. Acad. Sci. U. S. A. 117, 8669–8671. 10.1073/pnas.2002581117, PMID: 32269078 PMC7183192

[ref23] Bushnell W. R. (1967). “The dynamic role of molecular constituents in plant-parasite interactions,” in Symptom Development in Mildewed and Rusted Tissue. eds. Mirocha C. J. Uritani I. (St. Paul, MN: Bruce Publishing Company), 21–39.

[ref24] Chanclud E. Kisiala A. Emery N. R. Chalvon V. Ducasse A. Romiti-Michel C.. (2016). Cytokinin production by the rice blast fungus is a pivotal requirement for full virulence. PLoS Path. 12:2. 10.1371/journal.ppat.1005457 PMC4765853 26900703

[ref25] Chen L. Q. Qu X. Q. Hou B. H. Sosso D. Osorio S. Fernie A. R.. (2012). Sucrose efflux by SWEET proteins as a key step for pholem transport. Science 335, 207–211. 10.1126/science.1213351 22157085

[ref26] Cherniad’ev I. (2000). Ontogenetic changes in the photosynthetic apparatus and effects on cytokinins. Appl. Biochem. Microbiol. 36, 611–625. 10.1023/A:1026628119243 11116902

[ref27] Choi J. Huh S. U. Kojima M. Sakakibara H. Paek K. H. Hwang I. (2010). The cytokinin-activated transcription factor ARR2 promotes plant immunity via TGA3/NPR1-dependent salicylic acid signaling in *Arabidopsis*. Dev. Cell 19, 284–295. 10.1016/j.devcel.2010.07.011, PMID: 20708590

[ref28] Cordovez V. Schop S. Hordijk K. Dupré de Boulois H. Coppens F. Hanssen I.. (2018). Priming of plant growth promotion by volatiles of root-associated *Microbacterium* spp. Appl. Environ. Microbiol. 84:22. 10.1128/AEM.01865-18 PMC6210106 30194105

[ref29] Cortleven A. Leuendorf J. E. Frank M. Pezzetta D. Bolt S. Schmulling T. (2019). Cytokinin action in response to abiotic and biotic stresses in plants. Plant Cell Environ. 42, 998–1018. 10.1111/pce.13494, PMID: 30488464

[ref30] Criado M. V. Caputo C. Roberts I. N. Castro M. A. Barneix A. J. (2009). Cytokinin-induced changes of nitrogen remobilization and chloroplast ultrastructure in wheat (*Triticum aestivum*). J. Plant Physiol. 166, 1775–1785. 10.1016/j.jplph.2009.05.007, PMID: 19540618

[ref31] De Marco F. Batailler B. Thorpe M. R. Razan F. Le Hir R. Vilaine F.. (2021). Involvement of SUT1 and SUT2 sugar transporters in the impairment of sugar transport and changes in phloem exudate contents in *Phytoplasma*-infected plants. Int. J. Mol. Sci. 22:2. 10.3390/ijms22020745 PMC7828548 33451049

[ref32] Dekhuijzen H. M. (1980). The occurrence of free and bound cytokinins in clubroots and *Plasmodiophora brassicae*. Physiol. Plant. 49, 169–176. 10.1111/j.1399-3054.1980.tb02647.x 24258747

[ref33] Depuydt S. Dolezal K. Van Lijsebettens M. Moritz T. Holsters M. Vereecke D. (2008). Modulation of the hormone setting by *Rhodococcus fascians* results in ectopic KNOX activation in *Arabidopsis*. Plant Physiol. 146, 1267–1281. 10.1104/pp.107.113969, PMID: 18184732 PMC2259056

[ref34] Dhandapani P. Song J. C. Novak O. Jameson P. E. (2017). Infection by *Rhodococcus fascians* maintains cotyledons as a sink tissue for the pathogen. Ann. Bot. 119, 841–852. 10.1093/aob/mcw202, PMID: 27864224 PMC5378184

[ref35] Diaz C. Lemaitre T. Christ A. Azzopardi M. Kato Y. Sato F.. (2008). Nitrogen recycling and remobilization are differentially controlled by leaf senescence and development stage in *Arabidopsis* under low nitrogen nutrition. Plant Physiol. 147, 1437–1449. 10.1104/pp.108.119040, PMID: 18467460 PMC2442554

[ref36] Ditengou F. A. Müller A. Rosenkranz M. Felten J. Lasok H. Miloradovic van Doorn M.. (2015). Volatile signalling by sesquiterpenes from ectomycorrhizal fungi reprogrammes root architecture. Nat. Commun. 6:6279. 10.1038/ncomms7279 25703994 PMC4346619

[ref37] Ehness R. Roitsch T. (1997). Cooordinated induction of mRNAs for extracellular invertase and a glucose transporter in *Chenopodium rubrum* by cytokinins. Plant J. 11, 539–548. 10.1046/j.1365-313x.1997.11030539.x 9107040

[ref38] Evans J. L. Scholes J. D. (1995). How does clubroot alter the regulation of carbon metabolism in its host? Asp. Appl. Biol. 42, 125–132.

[ref39] Fife J. M. Price C. Fife D. C. (1962). Some properties of phloem exudate collected from root of sugar beet. Plant Physiol. 37, 791–792. 10.1104/pp.37.6.791, PMID: 16655730 PMC406248

[ref40] Fischer W.-N. Kwart M. Hummel S. Frommer W. B. (1995). Substrate specificity and expression profile of amino acid transporters (AAPs) in *Arabidopsis*. J. Biol. Chem. 270, 16315–16320. 10.1074/jbc.270.27.16315, PMID: 7608199

[ref41] Fischer W.-N. Loo D. D. F. Koch W. Ludewig U. Borrer K. J. Tegeder M.. (2002). Low and high affinity amino acid H+-cotransporters for cellular import of neutral and charged amino acids. Plant J. 29, 717–731. 10.1046/j.1365-313X.2002.01248.x, PMID: 12148530

[ref42] Gebauer P. Korn M. Engelsdorf T. Sonnewald U. Koch C. Voll L. M. (2017). Sugar accumulation in leaves of *Arabidopsis sweet11/sweet12* double mutants enhances priming of the salicylic acid-mediated defense response. Front. Plant Sci. 8:1378. 10.3389/fpls.2017.01378 28848581 PMC5550771

[ref43] Geiger D. R. Sheigh W.-J. (1993). Sink strength: learning to measure, measuring to learn. Plant Cell Environ. 16, 1017–1018. 10.1111/j.1365-3040.1996.tb02048.x

[ref44] Glanz-Idan N. Tarkowski P. Tureckova V. Wolf S. (2020). Root-shoot communication in tomato plants: cytokinin as a signal molecule modulating leaf photosynthetic activity. J. Exp. Bot. 71, 247–257. 10.1093/jxb/erz399, PMID: 31504736 PMC6913696

[ref45] Godt D. E. Roitsch T. (1997). Regulation and tissue-specific distribution of mRNAs for three extracellular invertase isoenzymes of tomato suggests an important function in establishing and maintaining sink metabolism. Plant Physiol. 115, 273–282. 10.1104/pp.115.1.273, PMID: 9306701 PMC158483

[ref46] Gohlke J. Deeken R. (2014). Plant responses to *Agrobacterium tumefaciens* and crown gall development. Front. Plant Sci. 5:155. 10.3389/fpls.2014.00155, PMID: 24795740 PMC4006022

[ref47] Grosskinsky D. K. Naseem M. Abdelmohsen U. R. Plickert N. Engelke T. Griebel T.. (2011). Cytokinins mediate resistance against *Pseudomonas syringae* in tobacco through increased antimicrobial phytoalexin synthesis independent of salicylic acid signaling. Plant Physiol. 157, 815–830. 10.1104/pp.111.182931, PMID: 21813654 PMC3192561

[ref48] Gunning B. E. S. Barkley W. K. (1963). Kinin-induced directed transport and senescence in detached oat leaves. Nature 199, 262–265. 10.1038/199262a0

[ref49] Gupta R. Pizarro L. Leibman-Markus M. Marash I. Bar M. (2020). Cytokinin response induces immunity and fungal pathogen resistance, and modulates trafficking of the PRR LeEIX2 in tomato. Mol. Plant Pathol. 21, 1287–1306. 10.1111/mpp.12978, PMID: 32841497 PMC7488468

[ref50] Gutiérez-Luna F. M. López-Bucio J. Altamiramo-Hernández J. Valencia-Cantero E. Reyes de la Cruz H. Macías-Rodríguez L. (2010). Plant growth-promoting rhizobacteria modulate root-system architecture in *Arabidopsis thaliana* through volatile organic compound emission. Symbiosis 51, 75–83. 10.1007/s13199-010-0066-2

[ref51] Hammes U. Z. Nielsen E. Honaas L. A. Taylor C. G. Schachtman D. P. (2006). AtCAT6, a sink-tissue-localized transporter for essential amino acids in *Arabidopsis*. Plant J. 48, 414–426. 10.1111/j.1365-313X.2006.02880.x, PMID: 17052324

[ref52] Hann D. R. Dominguez-Ferreras A. Motyka V. Dobrev P. I. Schornack S. Jehle A.. (2014). The *Pseudomonas* type III effector HopQ1 activates cytokinin signaling and interferes with plant innate immunity. New Phytol. 201, 585–598. 10.1111/nph.12544, PMID: 24124900

[ref53] Harms K. V. W. R. Schulz B. Frommer W. B. (1994). Isolation and characterization of P-type H+-ATPase genes from potato. Plant Mol. Biol. 26, 979–988. 10.1007/BF00028864, PMID: 8000010

[ref54] Hayes M. A. Davies C. Dry I. B. (2007). Isolation, functional characterization, and expression analysis of grapevine (*Vitis vinifera* L.) hexose transporters: differential roles in sink and source tissues. J. Exp. Biol. 58, 1985–1997. 10.1093/jxb/erm061 17452752

[ref55] Herbers K. Sonnewald U. (1998). Molecular determinants of sink strength. Curr. Opin. Plant Biol. 1, 207–216. 10.1016/S1369-5266(98)80106-4, PMID: 10066584

[ref56] Hirner A. Ladwig F. Stransky H. Okumoto S. Keinath M. Harms A.. (2006). *Arabidopsis* LHT1 is a high-affinity transporter for cellular amino acid uptake in both root epidermis and leaf mesophyll. Plant Cell 18, 1931–1946. 10.1105/tpc.106.041012, PMID: 16816136 PMC1533986

[ref57] Ho L. C. (1988). Metabolism and compartmentation of imported sugars in sink organs in relation to sink strength. Annu. Rev. Plant Physiol. Plant Mol. Biol. 39, 355–378. 10.1146/annurev.pp.39.060188.002035

[ref58] Hwang H. H. Wang M. H. Lee Y. L. Tsai Y. L. Li Y. H. Yang F. J.. (2010). *Agrobacterium*-produced and exogenous cytokinin-modulated *Agrobacterium*-mediated plant transformation. Mol. Plant Pathol. 11, 677–690. 10.1111/j.1364-3703.2010.00637.x, PMID: 20696005 PMC6640272

[ref145] Jarzyniak K. Banasiak J. Jamruszka T. Pawela A. Di Donato M. Novák O.. (2021). Early stages of legume–rhizobia symbiosis are controlled by ABCG-mediated transport of active cytokinins. Nat. Plants 7, 428–436. 10.1038/s41477-021-00873-6, PMID: 33753904

[ref59] Jian H. Lu K. Yang B. Wang T. Zhang L. Zhang A.. (2016). Genome-wide analysis and expression profiling of the SUC and SWEET gene families of sucrose transporters in oilseed rape (*Brassica napus* L.). Front. Plant Sci. 7:1464. 10.3389/fpls.2016.01464 27733861 PMC5039336

[ref146] Kalt-Torres W. Kerr P. S. Usuda H. Huber S. C. (1987). Diurnal changes in maize leaf photosynthesis. Plant Physiol. 83, 294–298e. 10.1104/pp.83.2.294 16665237 PMC1056349

[ref60] Kennedy I. R. (1966). Primary products of symbiotic nitrogen fixation II. Pulse-labelling of serradella nodules with 15N2. Acta. Biochim. Biophys. Acta. 130, 295–303. 10.1016/0304-4165(66)90224-8 5972844

[ref61] Kiba T. Kudo T. Kojima M. Sakakibara H. (2011). Hormonal control of nitrogen acquisition: roles of auxin, abscisic acid, and cytokinin. J. Exp. Bot. 62, 1399–1409. 10.1093/jxb/erq410, PMID: 21196475

[ref62] Kiba T. Naitou T. Koizumi N. Yamashino T. Sakakibara H. Mizuno T. (2005). Combinatorial microarray analysis revealing *Arabidopsis* genes implicated in cytokinin responses through the His->Asp phosphorelay circuitry. Plant Cell Physiol. 46, 339–355. 10.1093/pcp/pci033, PMID: 15695462

[ref63] Kieber J. J. Schaller G. E. (2018). Cytokinin signaling in plant development. Development 145:dev149344. 10.1242/dev.149344 29487105

[ref64] Ko K. W. Okada K. Koga J. Jikumaru Y. Nojiri H. Yamane H. (2010). Effects of cytokinin on production of diterpenoid phytoalexins in rice. J. Pestic. Sci. 35, 412–418. 10.1584/jpestics.G09-63

[ref65] Kouchi H. Yoneyama T. (1984). Dynamics of carbon photosynthetically assimilated in nodulated soy bean plants under steady-state conditions. 2. The incorporation of 13C into carbohydrates, organic acids, amino acids, and some storage compounds. Ann. Bot. 53, 883–896. 10.1093/oxfordjournals.aob.a086758

[ref66] Kutáček M. Rovenská J. (1991). Auxin synthesis in *Agrobacterium-tumefaciens* and *A. tumefaciens*-transformed plant-tissue. Plant Growth Regul. 10, 313–327. 10.1007/BF00024591

[ref67] Lara M. E. B. Garcia M. C. G. Fatima T. Ehness R. Lee T. K. Proels R.. (2004). Extracellular invertase is an essential component of cytokinin-mediated delay of senescence. Plant Cell 16, 1276–1287. 10.1105/tpc.018929, PMID: 15100396 PMC423215

[ref68] Lee C. W. Efetova M. Englemann J. C. Kramell R. Wasternack C. Ludwig-Miller J.. (2009). *Argrobacterium tumefaciens* promotes tumor induction by modulating pathogen defense in *Arabidopsis thaliana*. Plant Cell 21, 2948–2962. 10.1105/tpc.108.064576, PMID: 19794116 PMC2768927

[ref69] Lee D. J. Park J. Y. Ku S. J. Ha Y. M. Kim S. Kim M. D.. (2007). Genome-wide expression profiling of ARABIDOPSIS RESPONSE REGULATOR 7(ARR7) overexpression in cytokinin response. Mol. Gen. Genomics. 277, 115–137. 10.1007/s00438-006-0177-x, PMID: 17061125

[ref70] Lemaitre T. Gaufichon L. Boutet-Mercey S. Christ A. Masclaux-Daubresse C. (2008). Enzymatic and metabolic diagnostic of nitrogen deficiency in *Arabidopsis thaliana* Wassileskija accession. Plant Cell Physiol. 49, 1056–1065. 10.1093/pcp/pcn081, PMID: 18508804

[ref71] Lemoine R. La Camera S. Atanassova R. Deedaldeechamp F. Allario T. Pourtau N.. (2013). Source-to-sink transport of sugar and regulation by environmental factors. Front. Plant Sci. 4:272. 10.3389/fpls.2013.00272, PMID: 23898339 PMC3721551

[ref72] Lemonnier P. Gaillard C. Veillet F. Verbeke J. Lemoine R. Coutos-Thevenot P.. (2014). Expression of *Arabidopsis* sugar transport protein STP13 differentially affects glucose transport activity and basal resistance to *Botrytis cinerea*. Plant Mol. Biol. 85, 473–484. 10.1007/s11103-014-0198-5, PMID: 24817131

[ref73] Leopold A. C. Kawase M. (1964). Benzladenine effects on bean leaf growth and senscence. Am. J. Bot. 51, 294–298. 10.1002/j.1537-2197.1964.tb06633.x

[ref74] Liu G. Ji Y. Bhuiyan N. H. Pilot G. Selvaraj G. Zou J.. (2010). Amino acid homeostasis modulates salicylic acid-associated redox status and defense responses in *Arabidopsis*. Plant Cell 22, 3845–3863. 10.1105/tpc.110.079392, PMID: 21097712 PMC3015111

[ref75] Liu S. T. Kado C. I. (1979). Indoleacetic acid production: a plasmid function of *Agrobacterium tumefaciens* C58. Biochem. Biophys. Res. Commun. 90, 171–178. 10.1016/0006-291X(79)91605-X, PMID: 496970

[ref76] López-Carbonell M. A. Nadal M. (1998). Change in cell ultrastructure and zeatin riboside concentrations in *Hedera helix*, *Pelargonium zonale*, *Prunus avium*, and *Rubus ulmifolius* leaves infected by fungi. Plant Dis. 82, 914–918. 10.1094/PDIS.1998.82.8.914, PMID: 30856921

[ref77] Malagoli P. Laine P. Rossato L. Ourry A. (2005). Dynamics of nitrogen uptake and mobilization in field-grown winter oilseed rape (*Brassica napus*) from stem extension to harvest. I. Global N flows between vegetative and reproductive tissues in relation to leaf fall and their residual N. Ann. Bot. 95, 853–861. 10.1093/aob/mci091, PMID: 15701662 PMC4246740

[ref78] Malinowski R. Novak O. Borhan M. H. Spichal L. Strnad M. Rolfe S. A. (2016). The role of cytokinins in clubroot disease. Eur. J. Plant Pathol. 145, 543–557. 10.1007/s10658-015-0845-y

[ref79] Marella H. H. Nielsen E. Schachtman D. P. Taylor C. G. (2013). The amino acid permeases AAP3 and AAP6 are involved in root-knot nematode parasitism of *Arabidopsis*. Mol. Plant-Microbe Interact. 26, 44–54. 10.1094/MPMI-05-12-0123-FI, PMID: 23194341

[ref80] Masclaux-Daubresse C. Daniel-Vedele F. Dechorgnat J. Chardon F. Gaufichon L. Suzuki A. (2010). Nitrogen uptake, assimilation and remobilization in plants: challenges for sustainable and productive agriculture. Ann. Bot. 105, 1141–1157. 10.1093/aob/mcq028, PMID: 20299346 PMC2887065

[ref81] Mauch-Mani B. Baccelli I. Luna E. Flors V. (2017). Defense priming: an adaptive part of induced resistance. Annu. Rev. Plant Biol. 68, 485–512. 10.1146/annurev-arplant-042916-041132, PMID: 28226238

[ref82] McCormick A. J. Cramer M. D. Watt D. A. (2006). Sink strength regulates photosynthesis in sugarcane. New Phytol. 171, 759–770. 10.1111/j.1469-8137.2006.01785.x, PMID: 16918547

[ref83] Merewitz E. B. Du H. Yu W. Liu Y. Gianfagna T. Huang B. (2012). Elevated cytokinin content in ipt transgenic creeping bentgrass promotes drought tolerance through regulating metabolite accumulation. J. Exp. Biol. 15, 1315–1328. 10.1093/jxb/err372 PMC3276099 22131157

[ref84] Miller C. O. Skoog F. Okumura F. S. Von Saltza M. H. Strong F. M. (1956). Isolation, structure and synthesis of kinetin, a substance promoting cell division. J. Am. Chem. Soc. 78, 1375–1380. 10.1021/ja01588a032

[ref85] Miyawaki K. Matsumoto-Kitano M. Kakimoto T. (2004). Expression of cytokinin biosynthetic isopentenyltransferase genes in *Arabidopsis*: tissue specificity and regulation by auxin, cytokinin, and nitrate. Plant J. 37, 128–138. 10.1046/j.1365-313X.2003.01945.x, PMID: 14675438

[ref86] Mok D. W. S. Mok M. C. (2001). Cytokinin Metabolism and Action. Annu. Rev. Plant Physiol. Plant Mol. Biol. 52, 89–118. 10.1146/annurev.arplant.52.1.89, PMID: 11337393

[ref87] Mothes K. Engelbrecht L. (1961). Kinetin-induced directed transport of substances in excised leaves in the dark. Phytochemistry 1, 58–62. 10.1016/S0031-9422(00)82812-5

[ref88] Mothes K. Engelbrecht L. (1963). On the activity of kinetin-like root factor. Life Sci. 2, 852–857. 10.1016/0024-3205(63)90098-5

[ref89] Naseem M. Philippi N. Hussain A. Wangorsch G. Ahmed N. Dandekar T. (2012). Integrated systems view on networking by hormones in *Arabidopsis* immunity reveals multiple crosstalk for cytokinin. Plant Cell 24, 1793–1814. 10.1105/tpc.112.098335, PMID: 22643121 PMC3442570

[ref90] Nasholm T. Kielland K. Ganeteg U. (2009). Uptake of organic nitrogen by plants. New Phytol. 182, 31–48. 10.1111/j.1469-8137.2008.02751.x, PMID: 19210725

[ref91] Ninan A. S. Grant J. Song J. C. Jameson P. E. (2019). Expression of genes related to sugar and amino acid transport and cytokinin metabolism during leaf development and senescence in *Pisum sativum* L. Plants 8:3. 10.3390/plants8030076 PMC6473372 30934599

[ref92] Noiraud N. Maurousset L. Lemoine R. (2001). Transport of polyols in higher plants. Plant Physiol. Biochem. 39, 717–728. 10.1016/S0981-9428(01)01292-X

[ref93] Okazaki K. Kabeya Y. Suzuki K. Mori T. Ichikawa T. Matsui M.. (2009). The PLASTID DIVISION1 and 2 components of the chloroplast division machinery determine the rate of chloroplast division in land plant cell differentiation. Plant Cell 21, 1769–1780. 10.1105/tpc.109.067785, PMID: 19567705 PMC2714929

[ref94] Okumoto S. Koch W. Tegeder M. Fischer W. N. Biehl A. Leister D.. (2004). Root phloem-specific expression of the plasma membrane amino acid proton co-transporter AAP3. J. Exp. Bot. 55, 2155–2168. 10.1093/jxb/erh233, PMID: 15361541

[ref95] Pan Q. N. Cui B. M. Deng F. Y. Quan J. L. Loake G. J. Shan W. X. (2016). RTP1 encodes a novel endoplasmic reticulum (ER)-localized protein in *Arabidopsis* and negatively regulates resistance against biotrophic pathogens. New Phytol. 209, 1641–1654. 10.1111/nph.13707, PMID: 26484750

[ref96] Paul M. J. Foyer C. H. (2001). Sink regulation of photosynthesis. J. Exp. Bot. 52, 1383–1400. 10.1093/jexbot/52.360.1383, PMID: 11457898

[ref97] Peet M. M. Kramer P. J. (1980). Effects of decreasing source/sink ratio in soybeans on photosynthesis, photorespiration, transpiration, and yield. Plant Cell Environ. 2, 201–206. 10.1111/1365-3040.ep11581547

[ref98] Raggi V. (1974). Free and protein amino acids in the pustules and surrounding tissues of rusted bean. Phytopathol. Z. 81, 289–300. 10.1111/j.1439-0434.1974.tb02804.x

[ref99] Raggi V. (1976). Amino acids in mycelium of *Sphaerotheca pannosa* var. *persicae* and in the infected and surrounding tissues of peach leaves. Phytopathol. Mediterr. 15, 110–114.

[ref100] Reinders A. Sivitz A. B. Ward J. M. (2012). Evolution of plant sucrose uptake transporters. Front. Plant Sci. 3:22. 10.3389/fpls.2012.00022, PMID: 22639641 PMC3355574

[ref101] Ruan Y. L. (2014). Sucrose metabolism: gateway to diverse carbon use and sugar signaling. Annu. Rev. Plant Biol. 65, 33–67. 10.1146/annurev-arplant-050213-040251, PMID: 24579990

[ref102] Ruan Y. L. Jin Y. Yang Y. J. Li G. J. Boyer J. S. (2010). Sugar input, metabolism, and signaling mediated by invertase: roles in development, yield potential, and response to drought and heat. Mol. Plant 3, 942–955. 10.1093/mp/ssq044, PMID: 20729475

[ref103] Ryu C.-M. Farag M. A. Hu C.-H. Reddy M. S. Wei H.-X. Paré P. W.. (2003). Bacterial volatiles promote growth in *Arabidopsis*. Proc. Natl. Acad. Sci. U. S. A. 100, 4927–4932. 10.1073/pnas.0730845100 12684534 PMC153657

[ref104] Schwachtje J. Fischer A. Erban A. Kopka J. (2018). Primed primary metabolism in systemic leaves: a functional systems analysis. Sci. Rep. 8:216. 10.1038/s41598-017-18397-5 29317679 PMC5760635

[ref105] Schwachtje J. Whitcomb S. J. Firmino A. A. P. Zuther E. Hincha D. K. Kopka J. (2019). Induced, imprinted, and primed responses to changing environments: does metabolism store and process information? Front. Plant Sci. 10:106. 10.3389/fpls.2019.00106 30815006 PMC6381073

[ref106] Schwelm A. Fogelqvist J. Knaust A. Julke S. Lilja T. Bonilla-Rosso G.. (2015). The *Plasmodiophora brassicae* genome reveals insights in its life cycle and ancestry of chitin synthases. Sci. Rep. 5:11153. 10.1038/srep11153 26084520 PMC4471660

[ref107] Seifikalhor M. Aliniaeifard S. Hassani B. Niknam V. Lastochkina O. (2019). Diverse roles of gamma aminobutyric acid in dynamic plant cell responses. Plant Cell Rep. 38, 847–867. 10.1007/s00299-019-02396-z, PMID: 30739138

[ref108] Siddique S. Radakovic Z. S. De La Torre C. M. Chronis D. Novak O. Ramireddy E.. (2015). A parasitic nematode releases cytokinin that controls cell division and orchestrates feeding site formation in host plants. Proc. Natl. Acad. Sci. U. S. A. 112, 12669–12674. 10.1073/pnas.1503657112, PMID: 26417108 PMC4611629

[ref109] Skoog F. Miller C. O. (1957). Chemical regulation of growth and organ formation in plant tissues cultured *in vitro*. Symp. Soc. Exp. Biol. 11, 118–130. 13486467

[ref110] Smith A. M. Stitt M. (2007). Coordination of carbon supply and plant growth. Plant Cell Environ. 30, 1126–1149. 10.1111/j.1365-3040.2007.01708.x, PMID: 17661751

[ref111] Song J. Jiang L. Jameson P. E. (2015). Expression patterns of *Brassica napus* genes implicate IPT, CKX, sucrose transporter, cell wall invertase, and amino acid permease gene family members in leaf, flower, silique, and seed development. J. Exp. Bot. 66, 5067–5082. 10.1093/jxb/erv133, PMID: 25873685 PMC4513924

[ref112] Sonnewald U. Fernie A. R. (2018). Next-generation strategies for understanding and influencing source-sink relations in crop plants. Curr. Opin. Plant Biol. 43, 63–70. 10.1016/j.pbi.2018.01.004, PMID: 29428477

[ref113] Spallek T. Gan P. Kadota Y. Shirasu K. (2018). Same tune, different song - cytokinins as virulence factors in plant-pathogen interactions? Curr. Opin. Plant Biol. 44, 82–87. 10.1016/j.pbi.2018.03.002, PMID: 29555490

[ref114] Stes E. Francis I. Petry I. Dolzblasz A. Depuydt S. Vereechke D. (2013). The leafy gall syndrome induced by *Rhodococcus fascians*. FEMS Microbiol. Lett. 342, 187–195. 10.1111/1574-6968.12119, PMID: 23480693

[ref115] Sturm A. Tang G.-Q. (1999). The sucrose-cleaving enzymes of plants are crucial for development, growth and carbon partitioning. Trends Plant Sci. 4, 401–407. 10498964 10.1016/s1360-1385(99)01470-3

[ref116] Su Y. H. Frommer W. B. Ludewig U. (2004). Molecular and functional characterization of a family of amino acid transporters from *Arabidopsis*. Plant Physiol. 136, 3104–3113. 10.1104/pp.104.045278, PMID: 15377779 PMC523371

[ref117] Tarkowiski L. Signorelli S. Hofte M. (2020). Gamma-aminobutyric acid and related amino acids in plant immune responses: emerging mechanisms of action. Plant Cell Environ. 43, 1103–1116. 10.1111/pce.13734 31997381

[ref118] Tegeder M. (2014). Transporters involved in source to sink partitioning of amino acids and ureides: opportunities for crop improvement. J. Exp. Biol. 65, 1865–1878. 10.1093/jxb/eru012 24489071

[ref119] Tegeder M. Hammes U. Z. (2018). The way out and in: phloem loading and unloading of amino acids. Curr. Opin. Plant Biol. 43, 16–21. 10.1016/j.pbi.2017.12.002 29278790

[ref120] Tegeder M. Masclaux-Daubresse C. (2018). Source and sink mechanisms of nitrogen transport and use. New Phytol. 217, 35–53. 10.1111/nph.14876, PMID: 29120059

[ref121] Tegeder M. Rentsch D. (2010). Uptake and partitioning of amino acids and peptides. Mol. Plant 3, 997–1011. 10.1093/mp/ssq047, PMID: 21081651

[ref122] Tegeder M. Rentsch D. Patrick J. W. (2011). “Organic carbon and nitrogen transporters,” in The Plant Plasma Membrane. eds. Murphy A. Schulz B. Peer W. (Berlin, Heidelberg: Springer), 331–352.

[ref123] Thorne J. H. Koller H. R. (1974). Influence of assimilate demand on photosynthesis, diffusive resistances, translocation, and carbohydrate levels of soybean leaves. Plant Physiol. 54, 201–207. 10.1104/pp.54.2.201, PMID: 16658860 PMC541531

[ref124] Turgeon R. (1989). The sink-source transition in leaves. Annu. Rev. Plant Physiol. Plant Mol. Biol. 40, 110–138. 10.1146/annurev.pp.40.060189.001003

[ref125] Vacheron J. Desbrosses G. Bouffaud M.-L. Touraine B. Moënne-Loccoz Y. Muller D.. (2013). Plant growth-promoting rhizobacteria and root system functioning. Front. Plant Sci. 4:356. 10.3389/fpls.2013.00356, PMID: 24062756 PMC3775148

[ref126] Veillet F. Gaillard C. Coutos-Thevenot P. La Camera S. (2016). Targeting the AtCWIN1 gene to explore the role of invertases in sucrose transport in roots and during *Botrytis cinerea* infection. Front. Plant Sci. 7:1899. 10.3389/fpls.2016.01899, PMID: 28066461 PMC5167757

[ref127] Walerowski P. Gündel A. Yahaya N. Truman W. Sobczak M. Olszak M.. (2018). Clubroot disease stimulates early steps of phloem differentiation and recruits SWEET sucrose transporters within developing galls. Plant Cell 30, 3058–3073. 10.1105/tpc.18.00283 30413655 PMC6354258

[ref128] Walters D. R. McRoberts N. Fitt B. D. L. (2008). Are green islands red herrings? Significance of green islands in plant interactions with pathogens and pests. Biol. Rev. 83, 79–102. 10.1111/j.1469-185X.2007.00033.x, PMID: 18093233

[ref129] Wang C. Liu Y. Li S. S. Han G. Z. (2015). Insights into the origin and evolution of the plant hormone signaling machinery. Plant Physiol. 167, 872–886. 10.1104/pp.114.247403 25560880 PMC4348752

[ref130] Weber H. Borisjuk L. Heim U. Sauer N. Wobus U. (1997). A role for sugar transporters during seed development: molecular characterization of a hexose and a sucrose carrier in fava bean seeds. Plant Cell 9, 895–908. 10.1105/tpc.9.6.895, PMID: 9212465 PMC156966

[ref131] White A. C. Rogers A. Rees M. Osborne C. P. (2016). How can we make plants grow faster? A source-sink perspective on growth rate. J. Exp. Bot. 67, 31–45. 10.1093/jxb/erv447 26466662

[ref132] Williams P. H. Keen N. T. Strandberg J. O. Mcnabola S. S. (1968). Metabolite synthesis and degradation during clubroot development in cabbage hypocotyls. Phytopathology 58, 921–928.

[ref133] Xiong D. Huang J. Peng S. Li Y. (2017). A few enlarged chloroplasts are less efficient in photosynthesis than a large population of small chloroplasts in *Arabidopsis thaliana*. Sci. Rep. 7:5782. 10.1038/s41598-017-06460-0 28720786 PMC5515944

[ref147] Yadav U. P. Ayre B. G. Bush D. R. (2015). Transgenic approaches to altering carbon and nitrogen partitioning in whole plants: assessing the potential to improve crop yields and nutritional quality. Front. Plant Sci. 6:275. 10.3389/fpls.2015.00275, PMID: 25954297 PMC4405696

[ref134] Yamada K. Saijo Y. Nakagami H. Takano Y. (2016). Regulation of sugar transporter activity for antibacterial defense in *Arabidopsis*. Science 354, 1427–1430. 10.1126/science.aah5692 27884939

[ref135] Yang H. Postel S. Kemmerling B. Ludewig U. (2014). Altered growth and improved resistance of *Arabidopsis* against *Pseudomonas syringae* by overexpression of the basic amino acid transporter AtCAT1. Plant Cell Environ. 37, 1404–1414. 10.1111/pce.12244, PMID: 24895758

[ref136] Yokoyama A. Yamashino T. Amano Y. Tajima Y. Imamura A. Sakakibara H.. (2007). Type-B ARR transcription factors, ARR10 and ARR12, are implicated in cytokinin-mediated regulation of protoxylem differentiation in roots of *Arabidopsis thaliana*. Plant Cell Physiol. 48, 84–96. 10.1093/pcp/pcl040, PMID: 17132632

[ref137] Zhang L. Tan Q. Lee R. Trethewy A. Lee Y. H. Tegeder M. (2010). Altered xylem-phloem transfer of amino acids affects metabolism and leads to increased seed yield and oil content in *Arabidopsis*. Plant Cell 22, 3603–3620. 10.1105/tpc.110.073833, PMID: 21075769 PMC3015121

[ref138] Zhang C. Turgeon R. (2018). Mechanisms of phloem loading. Curr. Opin. Plant Biol. 43, 71–75. 10.1016/j.pbi.2018.01.009, PMID: 29448176

[ref139] Zhang X. Y. Wang X. L. Wang X. F. Xia G. H. Pan Q. H. Fan R. C.. (2006). A shift of phloem unloading from symplasmic to apoplasmic pathway is involved in developmental onset of ripening in grape berry. Plant Physiol. 142, 220–232. 10.1104/pp.106.081430, PMID: 16861573 PMC1557625

[ref140] Zhang H. Xie X. Kim M.-S. Kornyeyev D. A. Holaday S. Paré P. W. (2008). Soil bacteria augment *Arabidopsis* photosynthesis by decreasing glucose sensing and abscisic acid levels in planta. Plant J. 56, 264–273. 10.1111/j.1365-313X.2008.03593.x, PMID: 18573192

[ref141] Zhu K. Zhou Q. Shen Y. Yan J. Xu Y. Wang Z.. (2020). Agronomic and physiological performance of an indica –japonica rice variety with a high yield and high nitrogen use efficiency. Crop Sci. 60, 1556–1568. 10.1002/csc2.20150

[ref001] Zimmermann M. H. Ziegler H. (1975). Mechanisms of phloem loading. in Encyclopedia of Plant Physiology. New series, eds. Loewus F. A. Tanner W. (Berlin, Hiedlberg: Springer), 480–503. PMID:

